# Targeting STAT3 in systemic lupus erythematosus and lupus nephritis: mechanisms, therapeutic advances, and structure-informed perspectives

**DOI:** 10.3389/fphar.2026.1854754

**Published:** 2026-06-04

**Authors:** Fangyu Yi, Xiao Tu, Ruxi Jin, Jiayue Zhou, Yayu Li, Haiyang Liao, Wanyue Xu, Yuan Yuan, Mengting Wu, Peng Bi, Jiazhen Yin

**Affiliations:** 1 Hangzhou Clinical College, Zhejiang Chinese Medical University, Hangzhou, Zhejiang, China; 2 Hangzhou Hospital of Traditional Chinese Medicine, Zhejiang Chinese Medical University, Hangzhou, Zhejiang, China; 3 Department of Nephrology (Key Laboratory of Kidney Disease Prevention and Control Technology), Hangzhou Hospital of Traditional Chinese Medicine, Hangzhou, Zhejiang, China

**Keywords:** domain-specific inhibition, lupus nephritis, small molecule therapeutics, STAT3, structure-informed perspectives, systemic lupus erythematosus

## Abstract

This review summarizes the mechanistic and therapeutic relevance of Signal Transducer and Activator of Transcription 3 (STAT3) in systemic lupus erythematosus (SLE) and lupus nephritis (LN), with an emphasis on how structural information can inform drug development. STAT3 integrates cytokine- and growth factor-driven JAK-STAT signaling, supports pathological T cell differentiation, B cell activation, and renal inflammation, and has therefore emerged as a potential therapeutic node in lupus. Current pharmacological evidence includes traditional Chinese medicine-derived compounds, repurposed or conventional immunomodulators, and clinically advancing JAK-STAT pathway inhibitors; however, most agents act through upstream or indirect modulation rather than direct STAT3 targeting. Structure-informed analyses of candidate compounds further suggest differential druggability across STAT3 domains, with the CCD and SH2 domain providing plausible binding surfaces, whereas the DBD remains comparatively inaccessible. Together, current evidence supports STAT3 as a mechanistically important and therapeutically tractable axis in SLE and LN, while highlighting the need for biochemical, cellular, and *in vivo* validation of domain-selective inhibitors, dual-domain engagement strategies, and degrader-based approaches.

## Graphical Abstract

**Figure:**
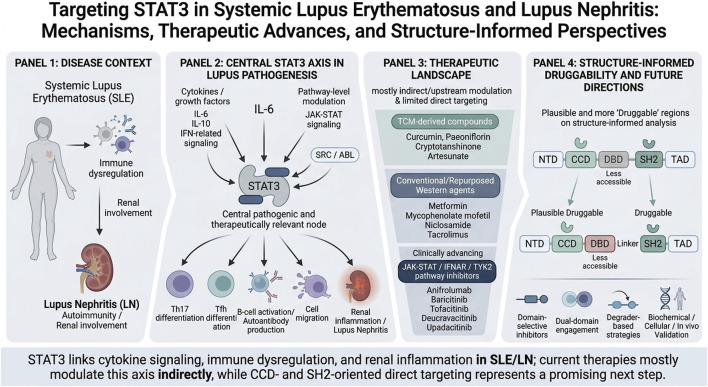

## 1 Introduction

Systemic lupus erythematosus (SLE) is a chronic autoimmune disease characterized by immune dysregulation, autoantibody production, and multi-organ damage. Lupus nephritis (LN), a severe renal complication of SLE, contributes substantially to disease-related morbidity and mortality. T cells play a critical role in SLE pathogenesis by supporting B-cell activation, producing inflammatory cytokines, and infiltrating target organs (Sammaritano et al., 2025). Among various signaling cascades, the Janus kinase/signal transducer and activator of transcription (JAK-STAT) pathway has emerged as a central mediator of immune abnormalities in SLE.

STAT proteins are central mediators of interferon (IFN)-driven immune signaling (Siegel and Sammaritano, 2024). More broadly, they transmit signals from over 50 cytokines, hormones, and growth factors, thereby regulating cellular survival, proliferation, and differentiation (Goropevšek et al., 2017). In SLE pathogenesis, dysregulated T cell activity contributes to disease by supporting B cells, producing inflammatory cytokines, and infiltrating target tissues (Bona et al., 2020). Within the JAK-STAT pathway, Signal Transducer and Activator of Transcription 3 (STAT3) serves as a key transcription factor downstream of multiple cytokines and growth factors (Jamilloux et al., 2019). Studies in both humans and lupus-prone mouse models have shown increased STAT3 expression and activation compared with healthy controls, supporting its involvement in autoimmune responses (Yoshida et al., 2019).

The aberrant activation of STAT3 in SLE affects immune responses in multiple ways. Given STAT3’s roles in B cell support, pathological T cell differentiation, and immune cell migration, modulation of this pathway has become a potential therapeutic strategy for SLE (Li et al., 2024). Together, these observations support STAT3 signaling as a viable therapeutic axis for SLE and its complications, particularly LN.

Evidence linking STAT3 to SLE and LN now spans mechanistic immunology, pharmacological intervention, clinical translation, and structural modeling. This review brings these strands together by summarizing the structural organization and regulatory mechanisms of STAT3, its functional relevance in SLE/LN immunopathogenesis, pharmacological modulation by Traditional Chinese Medicine (TCM)-derived and Western agents, and recent clinical advances in JAK-STAT/STAT3-related therapy. To complement this mechanistic and therapeutic overview, the review also considers STAT3 druggability from a structure-informed perspective, focusing on domain-level binding features that may guide future direct STAT3-targeted strategies.

## 2 Structural and regulatory basis of STAT3 relevant to lupus

**FIGURE 1:**
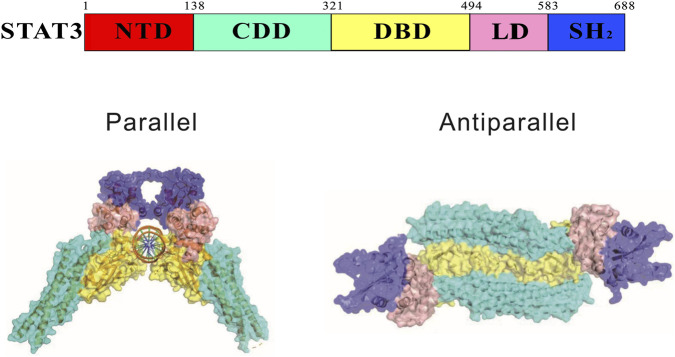
Parallel and antiparallel conformations of STAT3 dimers.

The human STAT3 gene is located on chromosome 17q21.1–17q21.2 and encodes a protein composed of 770 amino acids, with a molecular weight of approximately 88 kDa and a half-life of about 30 h. The STAT3 protein shuttles between the nucleus and cytoplasm and remains inactive in the cytoplasm when unphosphorylated. Upon phosphorylation, STAT3 translocates to the nucleus and becomes transcriptionally active. This hydrophilic protein contains six conserved functional domains: the N-terminal domain (NTD), coiled-coil domain (CCD), DNA-binding domain (DBD), linker domain, Src homology 2 (SH2) domain, and the transactivation domain (TAD) (Sgrignani et al., 2018). The NTD is a conserved sequence primarily responsible for the nuclear translocation of STAT3 dimers and subsequent DNA binding (Chalikonda et al., 2021). The CCD, composed of four α-helices connected by short loops, facilitates recruitment of STAT3 to receptors for phosphorylation (Chalikonda et al., 2021). The DBD recognizes specific DNA sequences, and the linker domain bridges the DBD and SH2 domains. The SH2 domain specifically recognizes phosphorylated tyrosine residues, mediating dimerization and playing a central role in intracellular signal transmission (Huang et al., 2020). The TAD includes two crucial phosphorylation sites, Tyr705 and Ser727, which are essential for STAT3 activation and transcriptional function. Studies have shown that targeting specific functional domains of STAT3, including the NTD, DBD, and SH2 domains, can effectively inhibit STAT3 activity (Wang X. et al., 2021; Chun et al., 2020; Liang et al., 2016). These structural features provide a foundation for understanding STAT3 as a therapeutically tractable target in SLE and LN (Figure 1).

The JAK-STAT signaling cascade is the most extensively characterized pathway for activating STAT3. Approximately 50 cytokines, including interleukin-6 (IL-6), interleukin-10 (IL-10), epidermal growth factor (EGF), fibroblast growth factor (FGF), and insulin-like growth factor (IGF), are known to activate this pathway (Kaur et al., 2020; Hunter and Jones, 2015). Upon ligand binding to their respective receptors, Janus kinases (JAKs) are activated and phosphorylate tyrosine residues on the receptor. STAT3 then binds to these phosphorylated sites via its SH2 domain and is subsequently phosphorylated by JAK. This results in the formation of homo- or heterodimers that translocate to the nucleus and mediate transcriptional regulation of genes involved in immune suppression, angiogenesis, metastasis, proliferation, and survival (Chen et al., 2019; Zhou et al., 2016). In addition to JAKs, non-receptor tyrosine kinases such as SRC and ABL can also activate STAT3 in the cytoplasm (Martellucci et al., 2020; Hassan et al., 2021). Together, these activation routes position STAT3 as a convergence point for cytokine and growth factor signals, a feature closely linked to immune dysregulation in SLE and LN.

Post-translational modifications (PTMs) further regulate STAT3 activity by influencing its phosphorylation status. Acetylation of lysine residues within the NH2- and SH2-domains enhances STAT3’s transcriptional potential, whereas histone deacetylases (HDACs) can suppress transcription of its target genes (Yang et al., 2021; Ortiz-Muñoz et al., 2010). Likewise, methylation and sumoylation are additional PTMs that critically modulate STAT3 function (Jiang et al., 2020). These PTMs add an additional layer of regulatory complexity and may influence the pharmacological tractability of STAT3.

Currently, three principal strategies are employed to inhibit the STAT3 signaling pathway. The first is direct inhibition, targeting its critical functional domains (NTD, DBD, or SH2) to block activation and dimerization (Chen et al., 2019; Estrada et al., 2018). The second strategy is upstream signal blockade, which involves inhibiting receptors or kinases that lead to STAT3 activation (Ren et al., 2021). The third approach involves immunomodulation, whereby immune regulators are manipulated to counteract pathogenic STAT3-mediated signaling. These strategies indicate that STAT3 can be pharmacologically addressed at multiple levels, from direct structural-domain blockade to pathway-level and immune-network modulation.

STAT3 plays a multifaceted role in SLE and its major complication, LN. It transduces signals through at least six classes of receptors and is particularly vital for T cell regulation. Conditional deletion of STAT3 in T cells has been shown to suppress the expression of IL-17 and IL-21, thereby reducing disease severity in autoimmune models (O'Shea et al., 2011; Murray, 2007). Th17 cells, major producers of IL-17 family cytokines, contribute to tissue injury in autoimmune diseases such as SLE by promoting neutrophil recruitment, enhancing B cell activation, and triggering pro-inflammatory cytokine production (Huang et al., 2022). These findings underscore STAT3’s central role in autoimmune pathogenesis, particularly through Th17 differentiation and inflammatory amplification (Kluger et al., 2016; Maeda et al., 2015). T cell-specific Stat3 deficiency in lupus-prone mice reduced lupus nephritis rather than only altering isolated cytokine readouts, indicating that STAT3-dependent T-cell programs can translate into renal disease expression (Yoshida et al., 2019). Future pharmacological studies should therefore report hard endpoints such as survival, proteinuria, renal histopathology, anti-dsDNA antibodies, and reversal of established nephritis, in addition to pSTAT3 or cytokine changes.

STAT3 is also essential for the differentiation of T follicular helper (Tfh) cells, which support B cell function in germinal centers (Zhang et al., 2023). Elevated expression of IL-21 and circulating Tfh-like cells have been observed in a wide array of human autoimmune diseases, including SLE (Zhu et al., 2016). Beyond Tfh biology, STAT3 also directly promotes B cell hyperresponsiveness in lupus. In B cells, STAT3 mediates downstream signaling of IL-6, IL-10, and IL-21, promoting antibody production via a STAT3-dependent mechanism (Alemán-García et al., 2021; Hedrich et al., 2014; Zhao et al., 2018). Global gene expression profiling has further revealed that aberrant STAT3 activation in B cells is associated with the Sle1ab genomic interval on mouse chromosome 1, which drives immune tolerance loss and antinuclear antibody (ANA) production in lupus-prone NZM2410 mice (Liu et al., 2005).

Recent studies indicate that T cells from SLE patients exhibit increased STAT3 phosphorylation, supporting its involvement downstream of chemokine signaling. Silencing STAT3 with small interfering RNA (siRNA) has confirmed its essential role in T cell migration in SLE (Ou et al., 2024). In lupus-prone MRL/lpr mice, pharmacologic inhibition of STAT3 using the small molecule inhibitor Stattic delayed the onset of autoantibody production and renal pathology, and also reduced lymphadenopathy and Tfh cell numbers, although Th17 cells remained unaffected (Edwards et al., 2015). These studies further support STAT3 not only as a mechanistic node but also as a preclinical target for intervention.

Given the central role of STAT3 in regulating immune responses and its relevance in SLE and LN, substantial efforts have been directed toward pharmacological agents capable of modulating STAT3 activity or its upstream signaling network. Advances in structural biology and molecular modeling have also enabled domain-oriented evaluation of compounds that may interact with STAT3 functional regions. Both TCM-derived and Western agents offer a diverse set of molecules with potential STAT3-axis-modulating properties. Building on this mechanistic foundation, the next section summarizes representative agents and their potential therapeutic significance in the context of SLE and LN.

## 3 JAK/STAT activation landscape in SLE and lupus nephritis

A broader JAK/STAT framework is needed when interpreting STAT3-directed therapy in SLE and LN. STAT proteins are transcription factors rather than kinases; therefore, treatment effects should be described as changes in STAT phosphorylation, nuclear translocation, DNA binding, or transcriptional activity. In SLE, STAT1 is closely linked to type I interferon and IFN-γ signatures, STAT3 integrates IL-6, IL-21, IL-23, Th17/Tfh differentiation and B-cell help, STAT4 reflects IL-12/IL-23 and TYK2-related signaling, and STAT5 is relevant to IL-2-dependent Treg homeostasis. Thus, JAK inhibition may reduce pSTAT3 while simultaneously altering pSTAT1, pSTAT4, or pSTAT5, making pathway-level pharmacodynamic profiling more informative than isolated pSTAT3 measurement (Goropevšek et al., 2017; Jamilloux et al., 2019).

Evidence from lupus models also supports a disease-specific role for STAT3. In particular, T cell-specific Stat3 deficiency abrogated lupus nephritis, linking STAT3-dependent T-cell programs to autoantibody formation, T-B-cell collaboration, renal immune-cell infiltration, and kidney injury in lupus rather than merely to generic autoimmune inflammation (Yoshida et al., 2019). This genetic evidence strengthens the biological rationale for STAT3 modulation, but it does not by itself prove that pharmacological STAT3 inhibition will reverse established nephritis or improve survival in MRL/lpr or NZB/W F1 models. Clinical JAK/TYK2 inhibitor studies further emphasize this non-selective biology. Baricitinib, tofacitinib, deucravacitinib, upadacitinib, and filgotinib act upstream of STAT proteins and affect interferon-, cytokine-, or TYK2-driven networks rather than STAT3 alone (Dörner et al., 2020; Dörner et al., 2022a; Dörner et al., 2022b; Yin et al., 2025; Hasni et al., 2021; Morand et al., 2023a; Mosca et al., 2025; Arriens et al., 2025; Merrill et al., 2025; Werth et al., 2022; Huo et al., 2023; Morand et al., 2024).

## 4 Pharmacological modulation of the STAT3 axis in SLE and lupus nephritis

STAT3-directed therapeutic exploration in SLE and LN spans natural products, TCM-derived components, conventional immunosuppressants, repurposed agents, and newer kinase inhibitors (Table 1). These agents do not represent a single pharmacological class. Instead, they affect the STAT3 axis at different levels, including suppression of JAK2/STAT3 phosphorylation, attenuation of IL-6/STAT3 signaling, modulation of Tfh/Th17 and B-cell responses, and reduction of oxidative stress and inflammatory amplification. The following sections summarize representative compounds supported by cellular, animal, mechanistic, or clinical evidence while preserving the distinction between direct STAT3 targeting and broader pathway modulation.

**TABLE 1:** Updated evidence-priority summary of recent and representative natural, synthetic, and clinical agents reported to modulate the STAT3/JAK axis in SLE and lupus nephritis.

Categories	Name	Mechanism	Ref.	Molecular structure
Natural agents	curcumin	Inhibition of the JAK2/STAT3 pathway	Gupta et al. (2013)	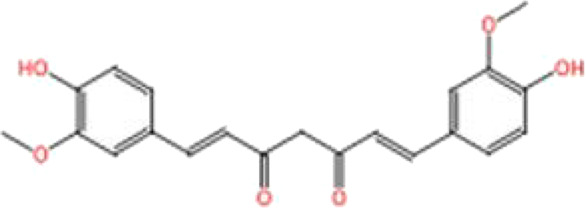
	Paeoniflorin (Pae)	Inhibition of the JAK2/STAT3 pathway	Zhang and Wei (2020)	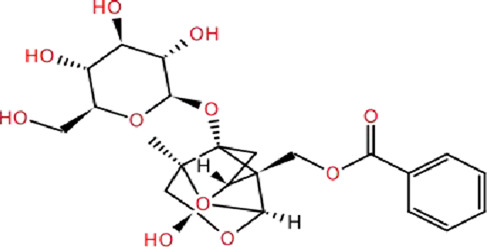
	Rutin	Reduce STAT3 activation	Yi et al. (2024)	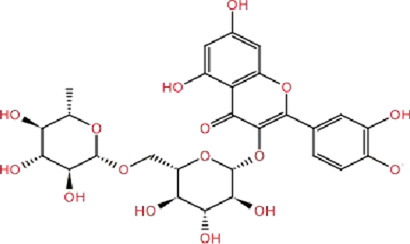
	Cryptotanshinone	Inhibit the activation of STAT3 both *in vivo* and *in vitro*	Du et al. (2019)	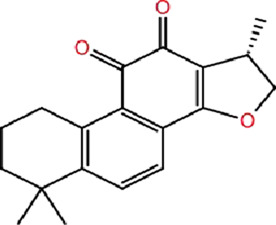
	Artesunate	Reduced the levels of phosphorylated JAK2 and STAT3	Dang et al. (2019)	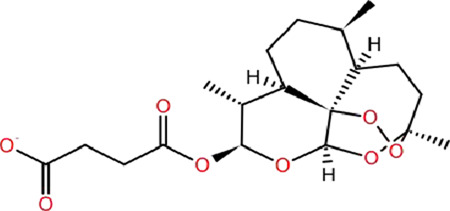
	Piperlongumine	Hampered the activation of JAK/STAT3 signaling in spleen cells	Yao et al. (2014)	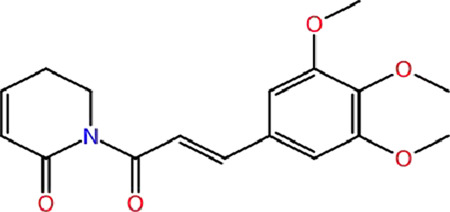
Natural derivatives	paeony (TGP)	Inhibition of the JAK2/STAT3 pathway	Zhang and Wei (2020)	​
	Hedyotis diffusa Willd	Significantly inhibits IL-6/STAT3	Xu et al. (2022)	​
	GYF-21	Downregulate the phosphorylation of NF-κB p65, Akt, and STAT3	Guo et al. (2019)	​
Synthetic agents	SM934	Hampered the comprehensive activation of STAT-1, STAT-3, and STAT-5 proteins in spleen cells	Hou et al. (2011)	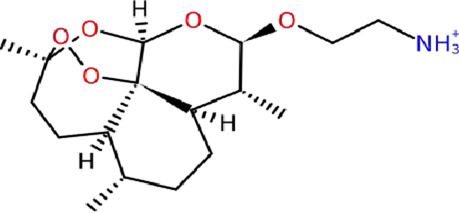
	Natura-α	Prevented the formation of STAT3 homodimers, thereby blocking the activation of STAT3	Chiao et al. (2016)	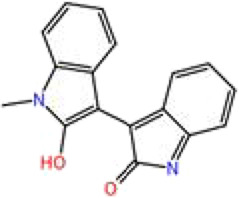
​	Metformin	Downregulated the expression of p-STAT3 through the AMPK/STAT3 pathway	Chen et al. (2022)	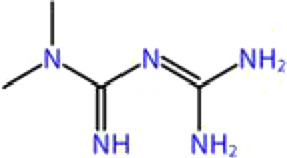
	Tofacitinib	Inhibit the phosphorylation of JAK-STAT to block the signaling pathway	Lin et al. (2022)	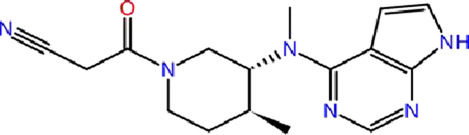
	Tacrolimus	Reduced the number of Th1, Th2, and Th17 cells, and combined treatment with STA-21 increased the number of Treg cells	Park et al. (2018)	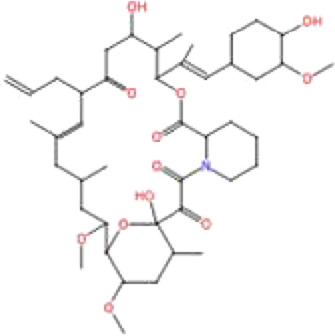
	Niclosamide	Lower the serum levels of IL-6 and IL-21	Jang et al. (2021)	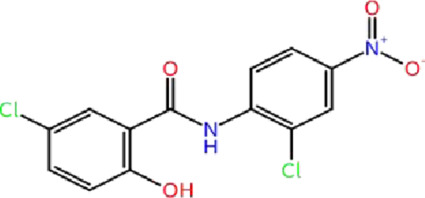
	Imiquimod	Inhibit the secretion of anti-dsDNA antibodies and anti-nuclear antibodies (ANA) in the serum	Duan et al. (2020)	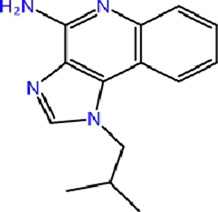
	Mycophenolate mofetil	Reduce p-STAT3 phosphorylation to inhibit the STAT3 pathway	Slight-Webb et al. (2019)	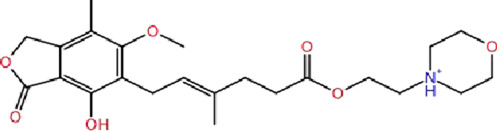
	CDDO-Me	Inhibit MEK-1/2, ERK, and STAT-3 signaling in lymphocytes and oxidative stress	Wu et al. (2014)	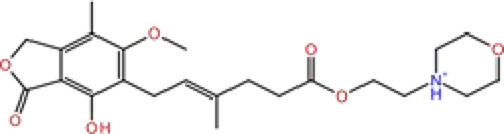
	Norcantharidin	Disrupt the specific activation of the IL-6/JAK/STAT3 signaling pathway	Du et al. (2022)	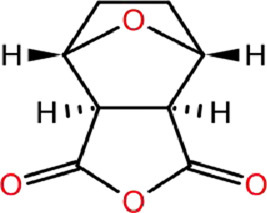
	Methyl salicylate 2-O-β-d-lactoside	Downregulate intracellular inflammatory signals NF-κB and JAK/STAT3	He et al. (2016)	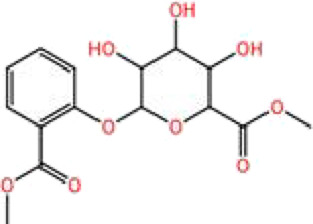
	Baricitinib	Simultaneous inhibition of IL-6 and Type I/II IFN signaling	Kubo et al. (2019)	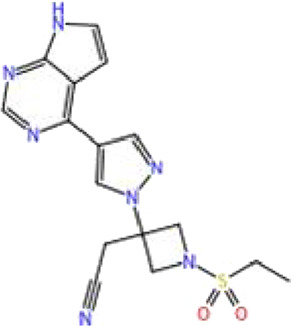
​	Cysteamine	Downregulate the IL-6/STAT-3 signaling pathway	Hsu et al. (2013)	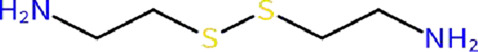
	Spermine	Inhibit JAK1 phosphorylation triggered by IFN-I, IFN-II, IL-2, and IL-6	Xu et al. (2024)	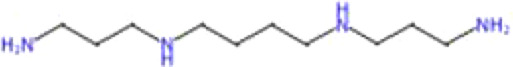
	Oleocanthal	Reduce immunoglobulin complex deposition, inflammation-mediated enzyme expression, and Th1/Th17 cytokine production	Montoya et al. (2023)	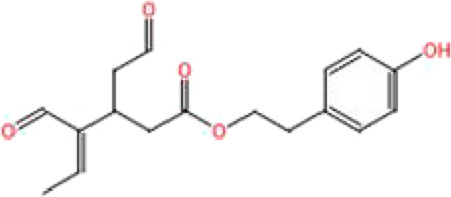
	Glucocorticoids	Increase the expression of STAT3 and IL-6R	P et al. (2011)	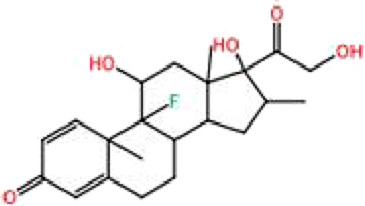
	Tapinarof	Inhibited the phosphorylation levels of JAK2 and STAT3	Zhang et al. (2023)	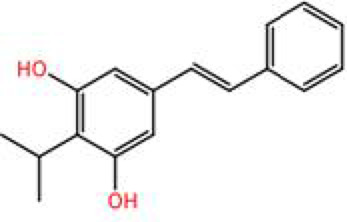
	Bardoxolone methyl	Inhibition of MEK-1/2, ERK, and STAT-3 signaling in lymphocytes and oxidative stress	Wu et al. (2014)	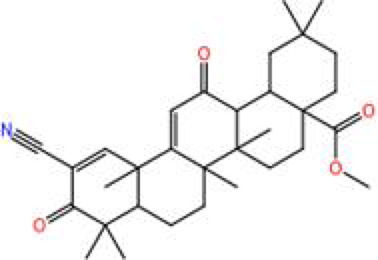
	GLPG3667	Dual inhibition of JAK1/TYK2	Mammoliti et al. (2024)	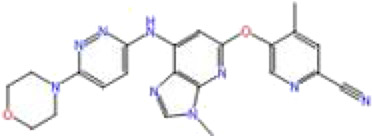

### 4.1 Natural agents

Recent PubMed-indexed systematic and mechanistic reviews of Chinese herbal medicine and natural-product approaches in SLE/LN support this evidence-priority framework (Wang Y. et al., 2021; Chen et al., 2023; Huang et al., 2025). To reduce repetitive citation with Table 1, the narrative highlights representative updated evidence: rutin has been linked to reduced T-cell oxidative stress in lupus nephritis (Yi et al., 2024), Hedyotis diffusa Willd was identified as an anti-inflammatory STAT3-related candidate in SLE (Xu et al., 2022), and oleocanthal improved renal and endothelial injury in pristane-induced SLE (Montoya et al., 2023). Additional agents with AMPK/STAT3, IL-6/JAK/STAT3, and JAK-linked immunometabolic signals, including metformin, norcantharidin, and spermine, are summarized in Table 1. Older compound-centered reports remain useful as background but should not be overinterpreted as evidence of direct STAT3-selective inhibition.

Curcumin, rutin, metformin, artesunate, piperlongumine, oleocanthal, and related compounds are highly pleiotropic and may influence AMPK, NF-κB, oxidative stress, PPARγ, Nrf2/HO-1, MAPK, and inflammasome pathways. A decrease in pSTAT3 after these interventions may therefore represent a downstream consequence of broader anti-inflammatory or metabolic effects rather than direct STAT3 inhibition, a caution that is consistent with recent natural-product evidence syntheses in lupus-related disease (Lubov et al., 2022).

### 4.2 Synthetic agents

#### 4.2.1 Selective or relatively direct STAT3 inhibitors

Among relatively direct STAT3 inhibitors or probes, the strongest lupus-relevant examples remain preclinical. Niclosamide has recent lupus-model evidence linking Tfh-cell suppression and disease improvement to STAT3 modulation (Jang et al., 2021), whereas Natura-α and STA-21-based strategies are better retained as classic mechanistic or target-engagement examples rather than as current translational evidence. This wording replaces the earlier overemphasis on older direct-inhibitor reports and separates mechanistic plausibility from therapeutic validation.

#### 4.2.2 Non-selective STAT3-associated or JAK/STAT modulators

A second subgroup consists of agents that reduce STAT3 activation indirectly or non-selectively. JAK inhibitors such as baricitinib, tofacitinib, upadacitinib, filgotinib, and TYK2 inhibition with deucravacitinib can modulate pSTAT3, but they also affect STAT1-, STAT4-, STAT5-, interferon-, and cytokine-related programs (Dörner et al., 2020; Dörner et al., 2022a; Dörner et al., 2022b; Yin et al., 2025; Hasni et al., 2021; Morand et al., 2023a; Mosca et al., 2025; Arriens et al., 2025; Merrill et al., 2025; Werth et al., 2022; Huo et al., 2023; Morand et al., 2024). Repurposed or conventional agents such as mycophenolate mofetil, metformin, bardoxolone methyl, spermine derivatives, and oleocanthal similarly influence broader metabolic, oxidative-stress, or immunoregulatory pathways (Montoya et al., 2023; Chen et al., 2022; Slight-Webb et al., 2019; Wu et al., 2014; Xu et al., 2024). Their effects should therefore be framed as STAT3-associated pathway modulation rather than direct STAT3-targeted therapy.

For synthetic and clinical agents, the evidence base was updated to prioritize recent JAK/TYK2 studies and clinically relevant pathway modulation. Baricitinib has mechanistic and clinical SLE evidence showing effects on immune gene-expression programs, anti-dsDNA responses, and pooled clinical outcomes (Dörner et al., 2020; Dörner et al., 2022a; Dörner et al., 2022b; Yin et al., 2025). Tofacitinib has randomized phase 1 safety data in SLE and additional clinical experience (Hasni et al., 2021; Ma et al., 2025; Yang et al., 2022; Bonnardeaux and Dutz, 2022; Sarkar et al., 2023). Deucravacitinib, an allosteric TYK2 inhibitor, has phase 2 SLE efficacy and patient-reported outcome data, with phase 3 studies underway (Morand et al., 2023a; Mosca et al., 2025; Arriens et al., 2025). Upadacitinib, filgotinib, and GLPG3667 further support the broader therapeutic relevance of upstream JAK/TYK2 modulation (Merrill et al., 2025; Werth et al., 2022; Mammoliti et al., 2024). These newer studies replace older synthetic-agent reports as the principal evidence for clinically maturing JAK/STAT-axis therapy.

Taken together, Direct or relatively selective STAT3 inhibitors remain largely preclinical in SLE/LN, whereas JAK/TYK2 inhibitors have stronger clinical evidence but are not STAT3-specific. Pleiotropic natural products and repurposed drugs should be considered STAT3-associated modulators unless direct binding, STAT3-selective rescue, or orthogonal target-engagement assays are available.

### 4.3 Recent clinical advances

Clinical translation has advanced most clearly through blockade at the IFNAR and JAK/TYK2 levels, whereas direct STAT3-targeted therapies for SLE and LN remain limited. Over the past 5 years, IFNAR1 blockade with anifrolumab has led clinical progress in JAK-STAT pathway modulation for SLE (Table 2). Phase 3 TULIP-1 and TULIP-2 trials provided evidence of clinical benefit across composite disease activity measures, with TULIP-2 meeting its primary endpoint (Furie et al., 2019; Morand et al., 2020). An open-label phase 2 study in Japanese patients confirmed acceptable safety and feasibility across Asian populations (Tanaka et al., 2020). Post hoc analyses from TULIP trials showed higher attainment of lupus low disease activity state (LLDAS) and sustained glucocorticoid tapering (Morand EF. et al., 2023; Bruce et al., 2023), and the long-term extension program supported durability of response and acceptable safety (Kalunian et al., 2023). Additional analyses further suggested benefit for patient-reported outcomes, remission or LLDAS attainment, organ-damage accrual, and refractory cutaneous disease (Strand et al., 2025; Morand et al., 2025; Touma et al., 2025; Baker et al., 2024; Miyazaki et al., 2024).

**TABLE 2:** Recent clinical advances in JAK-STAT/STAT3-related therapies in SLE and lupus nephritis.

Categories	Name	Target/Mechanism	Trial phase/Design	Population/Indication	Key outcomes
IFNAR1 blockade	Anifrolumab	Anti-IFNAR1 mAb; suppresses IFN-driven JAK-STAT signatures	Phase 3 RCTs (TULIP-1/2) + LTE (TULIP-LTE)	Active SLE	Primary endpoint met in TULIP-2; higher lupus low disease activity state (LLDAS) attainment and steroid tapering; durable patient-reported outcome (PRO) benefit
IFNAR1 blockade	Anifrolumab	Anti-IFNAR1 mAb; IFN pathway inhibition	Phase 2 RCT + extension	Active lupus nephritis	Renal response signal; maintenance into year 2; metabolomic modulation
JAK1/2 inhibitor	Baricitinib	JAK1/2 blockade	Phase 2 RCT; phase 3 pooled analysis	Active SLE	Downregulation of immune pathways; phase 3 efficacy mixed
JAK1/3 inhibitor	Tofacitinib	JAK1/3 blockade	Phase 1 randomized safety; real-world cohort; case reports	SLE; refractory cutaneous/bullous/alopecia	Safety/PD engagement; real-world disease control; case responses
Topical JAK inhibitor	Ruxolitinib (cream)	Local JAK1/2 inhibition	Case report	Cutaneous lupus lesions	Clinical improvement reported
TYK2 inhibitor	Deucravacitinib	Allosteric TYK2 inhibition	Phase 2 RCT; phase 3 ongoing	Active SLE	Efficacy + patient-reported outcome (PRO) improvements
JAK1-selective	Upadacitinib (with or without elsubrutinib)	Selective JAK1 inhibition	LTE through 104 weeks	SLE	Sustained responses
JAK1-selective	Filgotinib	Selective JAK1 inhibition	Phase 2 RCT	Moderate-to-severe cutaneous lupus	Efficacy signal
STAT3 node (indirect)	Mycophenolate mofetil	Reduces STAT3 phosphorylation	Mechanistic clinical evidence	SLE patients	Reduced p-STAT3

In lupus nephritis, a randomized phase 2 trial demonstrated that anifrolumab added to standard therapy yielded signals of improved renal response with manageable safety (Jayne et al., 2022). Second-year extension data suggested maintenance of renal benefit over time (Jayne et al., 2023), and metabolomic profiling indicated that IFN pathway inhibition modulates renal-associated metabolic signatures (Gavin et al., 2025).

For direct JAK inhibition, baricitinib (JAK1/2) has been evaluated in phase 2 SLE trials, with transcriptomic analyses indicating downregulation of multiple immune pathways (Dörner et al., 2020) and reductions in anti-dsDNA titers in clinical responders (Dörner et al., 2022a). Biomarker-focused analyses further supported its effects on interferon- and cytokine-driven networks (Dörner et al., 2022b), although pooled analyses from phase 3 SLE-BRAVE studies suggest mixed efficacy and highlight the need for refined patient stratification (Yin et al., 2025).

Tofacitinib (JAK1/3) has entered early-phase clinical evaluation; a phase one randomized trial in SLE demonstrated acceptable safety and pharmacodynamic engagement (Hasni et al., 2021). Real-world cohort data suggest potential disease-control benefits compared with glucocorticoids in mild-to-moderate SLE (Ma et al., 2025), and case-based reports describe responses in refractory bullous and cutaneous manifestations, including alopecia (Yang et al., 2022; Bonnardeaux and Dutz, 2022; Sarkar et al., 2023). Topical JAK inhibition (ruxolitinib) has also shown activity in cutaneous lupus lesions, supporting localized pathway targeting (Park et al., 2022).

TYK2 inhibition represents another clinically maturing strategy. Deucravacitinib demonstrated efficacy in a phase 2 randomized SLE trial (Morand et al., 2023a), with patient-reported outcome (PRO) improvements reinforcing clinical benefit (Mosca et al., 2025); phase 3 studies are now underway (Arriens et al., 2025). JAK1-selective approaches are also advancing: long-term extension data of upadacitinib (with or without elsubrutinib) indicate sustained responses through 104 weeks (Merrill et al., 2025), and filgotinib showed signals of efficacy in a phase 2 trial for moderate-to-severe cutaneous lupus (Werth et al., 2022).

Direct STAT3 inhibitors have not yet entered SLE or LN clinical trials, but human mechanistic evidence supports STAT3 as a therapeutic node; for example, mycophenolate mofetil reduced STAT3 phosphorylation in patients with SLE (Slight-Webb et al., 2019). Overall, clinical progress has been strongest for upstream JAK-STAT modulation, while direct STAT3-targeted therapy in SLE/LN remains an early translational goal that will require stronger pharmacodynamic and disease-relevant validation.

## 5 Safety and off-target considerations for STAT3 inhibition

STAT3 is not only a pathogenic transcription factor; it also contributes to epithelial repair, hematopoiesis, mucosal immunity, infection defense, and tissue regeneration. Sustained or systemic STAT3 inhibition may therefore carry risks that are particularly relevant in SLE, where patients often receive glucocorticoids, antimalarials, calcineurin inhibitors, mycophenolate, cyclophosphamide, biologics, or JAK inhibitors (Morand et al., 2023b; He and Li, 2023). Safety evaluation should include infection, cytopenia, hepatic and gastrointestinal toxicity, metabolic effects, reproductive considerations, and potential interference with protective IL-10- or IL-6-family cytokine responses.

Non-selective inhibition of other STAT family members is another major concern. Blocking upstream JAKs may suppress pSTAT1 and type I interferon signaling as well as pSTAT3, while JAK1/3 or TYK2-directed agents may also alter STAT4-or STAT5-dependent pathways (Huo et al., 2023; Morand et al., 2024). Even compounds described as STAT3 inhibitors may affect STAT1, STAT5, NF-κB, AMPK, oxidative-stress pathways, or mitochondrial function. Future studies should therefore measure pSTAT1, pSTAT3, pSTAT4, and pSTAT5 in parallel and use STAT3 knockdown/knockout or rescue experiments to establish specificity.

## 6 Structure-informed perspectives on STAT3 druggability

Because STAT3 contributes to Th17 differentiation, T follicular helper (Tfh) cell development, B cell activation, and renal inflammation, its domain-level architecture is relevant to therapeutic design in SLE and LN. Existing STAT3-targeted strategies suggest that small molecules or degrader-related ligands may influence downstream signaling by engaging functional regions such as the TAD, CCD, SH2 domain, and DBD. However, the accessibility, conformational context, and pharmacological relevance of these domains differ substantially.

**FIGURE 2:**
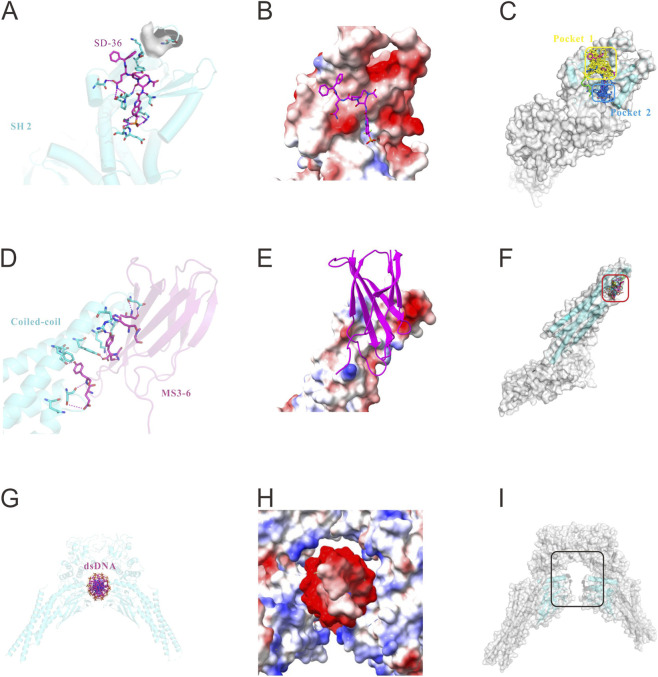
Structural modeling of STAT3 domain-specific interactions with small molecules and known ligands. **(A)** Interaction model of SD-36 and the SH2 domain of STAT3. **(B)** Electrostatic potential surface of the binding pocket between SD-36 and the SH2 domain. **(C)** Molecular docking of small molecules with the SH2 domain, with molecules occupying two pockets highlighted in yellow and blue. **(D)** Interaction model of MS3-6 and the CCD domain. **(E)** Electrostatic potential surface of the binding pocket between MS3-6 and the CCD domain. **(F)** Molecular docking of small molecules with the CCD domain. **(G)** Interaction model of dsDNA and the DBD domain. **(H)** Electrostatic potential surface of the binding interface between dsDNA and the DBD domain. **(I)** Molecular docking of small molecules with the DBD domain. The structural comparison focuses on binding regions through which ligands or inhibitors may influence STAT3-mediated signal transduction.

Previously reported candidate compounds from Natural agents, Natural derivatives, and conventional drugs were considered from a structure-informed perspective. Among 25 reported small molecules, 22 with retrievable structures were compared against the SH2, CCD, and DBD domains of STAT3 (Figures 2A–C). In the present review context, docking serves primarily as a comparative and hypothesis-generating tool for discussing STAT3 domain-level druggability, rather than as definitive validation of direct binding or functional inhibition (Stanzione et al., 2021).

### 6.1 Domain-oriented structural evaluation of candidate compounds

For the structural component of this review, comparative docking was used to place reported STAT3-axis-modulating compounds into a common domain-oriented framework. The analysis considered Natural agents, Natural derivatives, and synthetic or clinically used drugs with available structures. Docking was performed with AutoDock Vina v1.2.0 using small-molecule structures from PubChem and ZINC and a processed human STAT3 crystal structure from the Protein Data Bank (PDB ID: 6NUQ).

The comparison focused on three major STAT3 regions: the SH2 domain, coiled-coil domain (CCD), and DNA-binding domain (DBD). Domain-level grid boxes were used to orient docking around these regions, and the known ligands SD-36 and MS3-6 were included as structural reference controls for interpreting SH2- and CCD-oriented binding. Binding energy and spatial fit were considered as comparative indicators rather than as standalone evidence of biological activity.

PyMOL and Discovery Studio Visualizer were used to inspect hydrogen bonding, hydrophobic interactions, and pocket occupancy. The resulting interaction patterns were interpreted alongside available biological and pharmacological evidence, with the aim of identifying plausible domain-level preferences that may guide future validation. Thus, the structural assessment complements mechanistic and pharmacological evidence without replacing biochemical, cellular, or *in vivo* confirmation.

Accordingly, AutoDock Vina scores should not be used to claim domain-specific druggability or direct STAT3 binding. Predicted CCD-, SH2-, or DBD-oriented interactions require confirmation by orthogonal biophysical assays such as surface plasmon resonance or isothermal titration calorimetry (Krishnamoorthy et al., 2020), and by cell-based assays assessing STAT3 phosphorylation, dimerization, nuclear translocation, DNA binding, and transcriptional activity.

### 6.2 Differential druggability across major STAT3 domains

STAT3 comprises six functionally conserved domains, including the NTD, CCD, DBD, SH2 domain, linker domain, and TAD. Previous studies have shown that several of these domains, particularly the SH2 and CCD domains, are key regulatory sites involved in STAT3 activation and dimerization. Among these, the SH2 domain remains the most widely investigated interface for small molecule binding.

Available structural evidence also supports the therapeutic relevance of the CCD domain, which can interact with synthetic peptides such as SD-36 and has been linked to STAT3 degradation and downstream pathway modulation. This has expanded interest in CCD-oriented inhibitors or degraders that may disrupt STAT3 localization or regulatory interactions. These observations suggest that the CCD domain may be an underexplored but potentially druggable site for STAT3 modulation.

Comparative structural assessment of the 22 candidate compounds against the SH2, CCD, and DBD domains provided a domain-level view of potential binding preferences (Figures 2A–C). These patterns are best interpreted as structure-informed hypotheses, helping to compare TCM-derived monomers, their derivatives, and conventional synthetic drugs across the same STAT3 domain framework.

Available structural features of the DBD help explain why this region appears less favorable for small-molecule accommodation. The DBD, formed within the STAT3 homodimer and responsible for binding double-stranded DNA, is characterized by a strongly positively charged environment, hydrophobic features, and limited spatial accessibility (Figures 2G–I). These properties support the view that the DBD is a challenging site for conventional small-molecule targeting.

By contrast, the CCD domain provided a more favorable structural setting in the comparative assessment. Using the previously reported STAT3-MS3-6 peptide binding pocket as a reference, many candidate compounds could be accommodated within the CCD-oriented site (Figures 2D–F). This pattern supports the view that CCD-centered design, including degrader-oriented strategies, may deserve greater attention in future STAT3-targeted drug development.

### 6.3 Representative interaction patterns of domain-selective and dual-domain compounds

**FIGURE 3:**
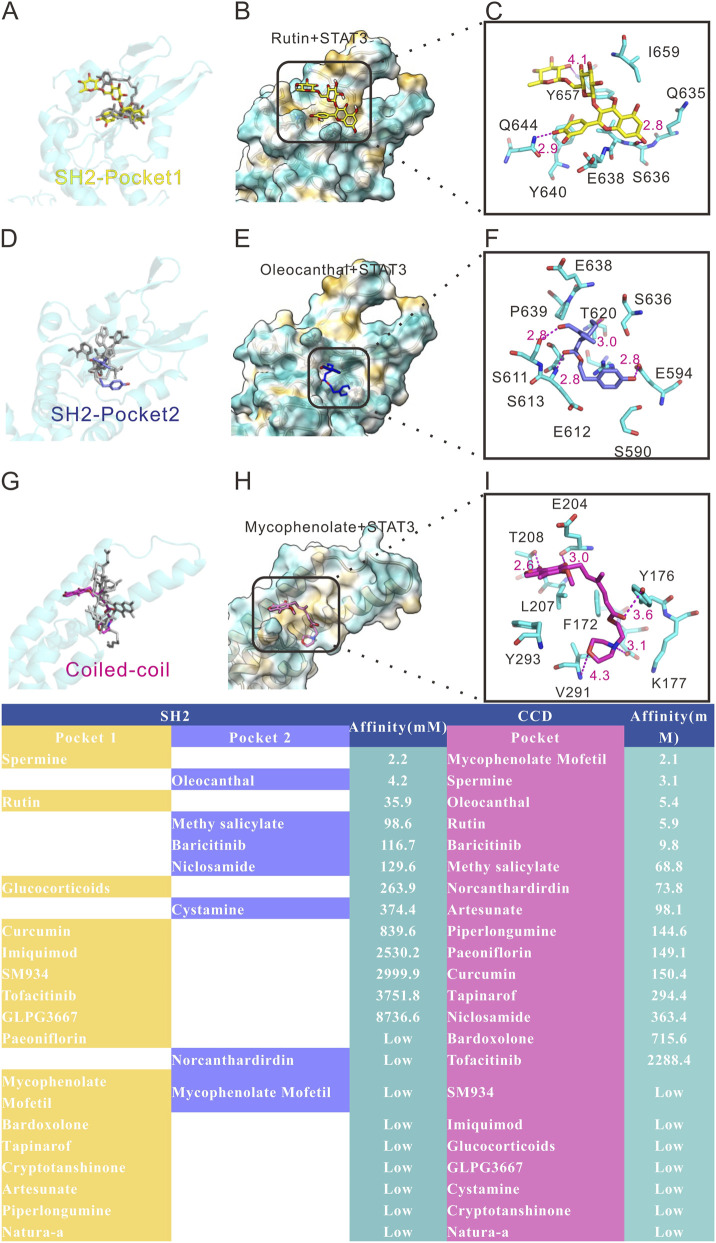
The affinity between STAT3 domains and small molecules. **(A)** Ribbon model showing Rutin occupying SH2 Pocket 1; **(B)** Surface model depicting Rutin binding in an acidic environment; **(C)** Detailed hydrogen bond interactions with residues such as Q635, E638, S636, and Y657; **(D)** Ribbon model showing Oleocanthal docking in Pocket 2; **(E)** Electrostatic surface of SH2 with Oleocanthal binding region highlighted; **(F)** Interaction map showing hydrogen bonding with E594, E638, E612, and S611; **(G)** Ribbon model showing Mycophenolate at the CCD pocket; **(H)** Hydrophobic surface representation with Mycophenolate enclosed in the CCD site; **(I)** Detailed interactions with E204, Y176, K177, and multiple stabilizing residues via hydrogen bonding. Bottom table: Summary of small molecule binding affinities (in μM) for the SH2 domain (Pockets 1 and 2) and CCD domain. Yellow and blue sections indicate molecules binding SH2 Pocket 1 and Pocket 2, respectively. The pink section indicates molecules binding the CCD pocket. “Low” indicates no measurable affinity or unstable interaction observed during docking.

Representative patterns include mycophenolate mofetil occupying a hydrophobic pocket within the CCD. In the model, its backbone was stabilized mainly by hydrophobic contacts, with hydrogen bonds at both molecular termini contributing to the predicted STAT3-ligand complex (Figures 3G–I). In the SH2 domain, the comparative structural assessment distinguished two pockets with different physicochemical environments. Pocket 1, characterized by an acidic surface, accommodated 15 compounds, among which Rutin showed the highest predicted affinity (∼36.0 μM) (Figures 3A–C). Pocket 2, enriched in basic residues, bound seven compounds, with Oleocanthal showing the strongest predicted affinity (∼4.2 μM) (Figures 3D–F). The comparatively stronger and more consistent values for Pocket 2 support the view that this region may be more amenable to ligand engagement than Pocket 1.

Across the assessed domains, the CCD showed the most consistent predicted interactions with small molecules. Mycophenolate mofetil, Spermine, Oleocanthal, Rutin, and Baricitinib displayed predicted affinities below 10 μM, followed by methyl salicylate, Norcantharidin, Artesunate, Piperlongumine, Paeoniflorin, Curcumin, Tapinarof, Niclosamide, and Bardoxolone, which also showed favorable CCD-oriented binding. Other molecules showed weaker predicted CCD affinity. The SH2 domain displayed a more heterogeneous pattern: Spermine, Oleocanthal, and Rutin showed stronger predicted SH2 binding, whereas methyl salicylate, Baricitinib, Niclosamide, glucocorticoids, cystamine, and Curcumin showed moderate affinity, particularly at Pocket 2. Thirteen additional compounds could occupy the SH2 binding site but with lower predicted affinities.

Several compounds, including Spermine, Oleocanthal, Rutin, methyl salicylate, Baricitinib, Niclosamide, and Curcumin, showed potential dual-domain engagement of the CCD and SH2 domains. Such patterns suggest that selected molecules might influence STAT3 signaling through more than one structural region, possibly depending on conformational or activation states of the protein. By contrast, glucocorticoids and cystamine were more SH2-selective in the comparison, while mycophenolate mofetil, Norcantharidin, Artesunate, Piperlongumine, Paeoniflorin, Tapinarof, and Bardoxolone were more CCD-oriented. These apparent selectivity patterns should be interpreted cautiously, but they may reflect differences in electrostatic complementarity, pocket geometry, and ligand physicochemical properties.

The interaction models provide a structural rationale for comparing historically studied SH2-directed strategies with the less explored CCD domain (Figure 3). These patterns support the view that both dual-domain and domain-selective compounds may be useful starting points for future STAT3-directed therapeutic design. They also broaden the design space beyond phosphorylation or dimerization blockade toward CCD targeting and degron-associated strategies, while underscoring the need for functional validation.

### 6.4 Pharmacological implications of structure-informed STAT3 targeting

These structure-informed observations provide a useful framework for understanding the domain-level druggability of STAT3 in SLE and LN. In the comparative assessment, the CCD showed the most consistent predicted interactions with small molecules, whereas the SH2 domain displayed more variable binding across two sub-pockets with distinct physicochemical environments. The DBD remained comparatively unfavorable for small-molecule accommodation, consistent with its charged and spatially constrained interface.

Potential dual-domain binders, including Spermine, Oleocanthal, Rutin, Niclosamide, and Curcumin, provide a rationale for exploring multitarget strategies that engage STAT3 at more than one regulatory region. Domain-selective patterns, such as mycophenolate mofetil at the CCD and cystamine at the SH2 domain, suggest that electrostatic complementarity and domain geometry may be useful design considerations. These interactions may be relevant to processes such as STAT3 dimerization, nuclear translocation, transcriptional activation, or degron-oriented targeting, but they require direct experimental confirmation.

**FIGURE 4:**
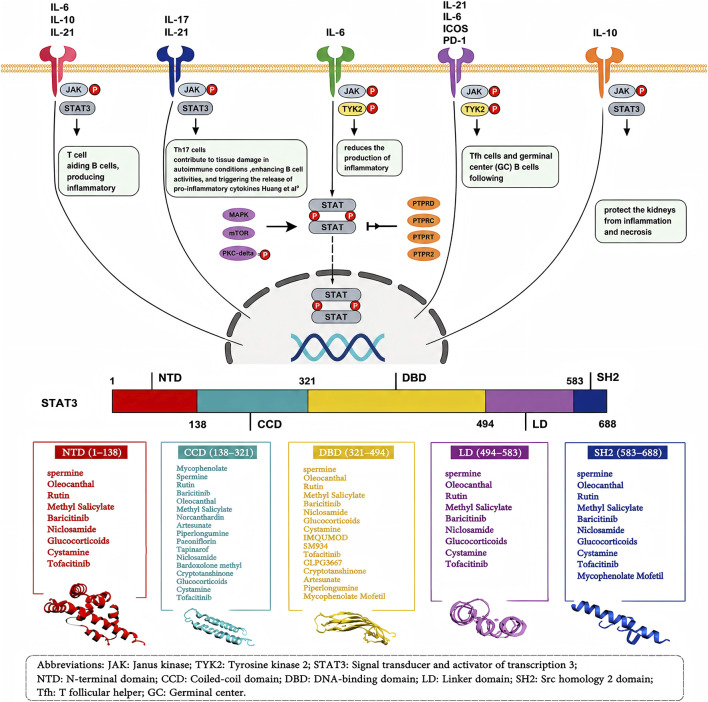
Domain-specific targeting strategies of small molecule inhibitors for STAT3 in SLE and lupus nephritis.

Importantly, this domain-level perspective reinforces the need to move beyond traditional phosphorylation-blocking approaches, including JAK inhibition alone. Combined-domain or degron-based strategies may offer routes toward improved selectivity, binding stability, and immunomodulatory efficacy. This structure-informed framework complements evidence from cytokine stimulation, Th17 and Tfh differentiation, and renal inflammation in SLE and LN (Figure 4).

The pharmacological implications of these observations should be interpreted with appropriate caution. Drug efficacy is not determined by static docking affinity alone, but also by target engagement in relevant conformational states, cellular pathway modulation, potential synergistic or antagonistic drug-drug interactions, and the capacity to reshape intracellular signaling networks.

## 7 Challenges and future directions

Several challenges still temper the translation of STAT3-directed strategies in SLE and LN. Most existing agents modulate the STAT3 axis indirectly through upstream cytokines, JAK/TYK signaling, oxidative-stress pathways, or broader immune reprogramming, rather than acting as direct STAT3-targeted agents. The evidence base is also uneven: many natural products and TCM-derived compounds remain supported mainly by cellular or animal studies, with limited pharmacokinetic, toxicity, and clinical validation.

Structure-informed inference, including docking-based prioritization, should therefore be viewed as hypothesis-generating rather than confirmatory. Candidate interactions require biochemical binding validation, cellular confirmation of STAT3 pathway modulation, and *in vivo* evidence of relevance to lupus immunopathogenesis and renal injury. Future development should emphasize domain-selective design, dual-domain engagement, degrader strategies, rational combination therapy, and precision stratification of lupus patients according to interferon, JAK-STAT, and STAT3-related immune signatures.

## 8 Conclusion

Taken together, the revised evidence hierarchy identifies STAT3 as a pathogenic and therapeutically relevant node in SLE and LN, but places the strongest current translational support at the level of upstream JAK/TYK2 or cytokine-pathway modulation rather than selective STAT3 blockade. Recent clinical and translational data for baricitinib, tofacitinib, deucravacitinib, upadacitinib, filgotinib, GLPG3667, and mycophenolate-associated pSTAT3 reduction support this more cautious framework (Dörner et al., 2020; Dörner et al., 2022a; Dörner et al., 2022b; Yin et al., 2025; Hasni et al., 2021; Morand et al., 2023a; Mosca et al., 2025; Arriens et al., 2025; Merrill et al., 2025; Werth et al., 2022; Slight-Webb et al., 2019; Ma et al., 2025; Yang et al., 2022; Bonnardeaux and Dutz, 2022; Sarkar et al., 2023; Mammoliti et al., 2024). Older small-molecule and natural-product studies remain useful for hypothesis generation, but they should not be used alone to claim direct STAT3-specific therapeutic efficacy.
